# Survival and Quality of Life After Reirradiation With Concurrent Pemetrexed/Erlotinib Followed by Maintenance Erlotinib for Recurrent/Second Primary Head and Neck Squamous Cell Carcinoma: Final Report of a Phase I/II Trial

**DOI:** 10.7759/cureus.106810

**Published:** 2026-04-10

**Authors:** Ryan T Hughes, Sydney Smith, D. Neil Hayes, Bhishamjit Chera, Ralph B D'Agostino Jr., Joshua D Waltonen, Christopher A Sullivan, James D Browne, Kathryn M Greven, Mercedes Porosnicu

**Affiliations:** 1 Radiation Oncology, Wake Forest University School of Medicine, Winston-Salem, USA; 2 Biostatistics and Data Science, Wake Forest University School of Medicine, Winston-Salem, USA; 3 Center for Cancer Research, The University of Tennessee Health Science Center, Memphis, USA; 4 Radiation Oncology, Medical University of South Carolina, Charleston, USA; 5 Otolaryngology - Head and Neck Surgery, Wake Forest University School of Medicine, Winston-Salem, USA; 6 Cancer Medicine, Wake Forest University School of Medicine, Winston-Salem, USA

**Keywords:** head and neck squamous cell carcinoma (hnscc), patient-reported outcome measures, quality of life, radiotherapy (rt), reirradiation

## Abstract

Introduction

For patients with recurrent/second primary (R/SP) head and neck squamous cell carcinoma (HNSCC) within a previously irradiated volume that is not amenable to surgical resection, reirradiation may be employed with curative intent. The optimal regimen for concurrent chemotherapy with reirradiation has not been determined. We sought to determine the maximum tolerated dose (MTD) of concurrent erlotinib in patients treated with definitive reirradiation, to determine disease response, PFS, and OS in patients treated with concurrent pemetrexed/erlotinib and maintenance erlotinib, and to describe quality of life (QOL) over time in this cohort.

Methods

A multi-institutional phase I/II clinical trial enrolled patients with R/SP HNSCC with disease within the 45 Gy isodose line. Patients were treated with concurrent pemetrexed/erlotinib followed by maintenance erlotinib. The MTD of concurrent erlotinib was determined using a 3+3 design. Adverse events (AEs) and patient-reported outcomes of QOL, including the Functional Assessment of Cancer Therapy-Head and Neck (FACT-HN) and MD Anderson Dysphagia Inventory (MDADI) and the Performance Status Scale for Head and Neck Cancer (PSS-HN), were collected at baseline, 1, 6, and 12 months after RT.

Results

In total, 27 patients were enrolled: 15 in phase I and 12 in phase II. Median prior RT dose was 66 Gy, and median reirradiation dose was 60 Gy. Two DLTs were observed in the 150 mg group; the MTD of erlotinib was 125 mg. Twenty-five of 27 patients experienced grade 3+ AEs. Best response was complete, partial, stable, and progressive in seven, seven, five, and four patients, respectively. Median PFS was 8.5 months, and median OS was 11.2 months. One-year PFS and OS rates were 33% and 44%, respectively. Mean FACT-HN trial outcome index ranged from 52 to 58 across all time points. Mean MDADI global and composite scores ranged from 57-75 to 56-66 over time, respectively. No differences in mean FACT-HN, MDADI, and PSS-HN scores were identified from baseline to six months.

Conclusions

In patients with R/SP HNSCC treated with reirradiation with concurrent pemetrexed/erlotinib followed by maintenance erlotinib, toxicity is high, disease control is consistent with prior reirradiation studies, and patient-reported QOL is unchanged within a short-term follow-up of six months.

## Introduction

Locoregional failure after treatment for de novo head and neck squamous cell carcinoma (HNSCC) is a significant occurrence experienced by 10-50% of patients [1,2]. Surgical management, which is often considered the optimal upfront approach for recurrent/second primary (R/SP) HNSCC, may not be feasible or acceptable [3]. In these cases, where patient, disease, and prior treatment factors allow, reirradiation with curative intent may be considered [3-7]. Reirradiation is associated with expected acute radiotherapy (RT) toxicities as well as several more severe risks that could substantially impact quality of life (QOL) [4,6,7]. Given these objective risks, the decision to proceed with reirradiation necessitates a nuanced discussion of expected benefits versus risks, particularly with regard to QOL [8]. Patient-reported outcomes (PROs) provide an additional perspective of global and disease-specific QOL when used in combination with clinician-rated outcomes, but few of the landmark trials for patients with R/SP HNSCC collected or reported PROs [4,7]. Additional focus on the collection and reporting of PRO measures in patients treated with reirradiation is needed [5].

Based on early translational and clinical data suggesting a radiosensitizing and antitumor activity for the combination of estimated Glomerular Filtration Rate (eGFR) inhibitors and pemetrexed, we conducted a phase I/II trial of reirradiation with concurrent pemetrexed/erlotinib followed by maintenance erlotinib for patients with R/SP HNSCC, hypothesizing that this combination would have a more favorable toxicity profile while maintaining efficacy [9-12]. The phase I component of this study aimed to determine the safety and maximum tolerated dose (MTD) of concurrent erlotinib with reirradiation. The phase II component sought to measure progression-free survival (PFS) and overall survival (OS) in a cohort of patients treated with the regimen identified in phase I. Both phases evaluated adverse events (AEs) and QOL outcomes in patients treated with reirradiation for R/SP HNSCC. In this final report, we describe the safety, response, survival, and QOL outcomes for the total cohort.

## Materials and methods

This multi-institutional phase I/II trial (CCCWFU 60107) enrolled adults with pathologically confirmed, measurable R/SP HNSCC who were ineligible for or declined surgical resection. Patients must have had prior RT (at least 45 Gy) completed at least six months prior to diagnosis of R/SP disease, Eastern Cooperative Oncology Group (ECOG) performance status 0-1, and adequate organ function. Exclusion criteria included metastatic disease, nasopharyngeal carcinoma, and uncontrolled intercurrent illness. All patients provided written informed consent; the study was approved by the Wake Forest University Health Sciences, Institutional Review Board (IRB00003457). This study was registered on ClinicalTrials.gov (NCT00573989).

Definitive reirradiation with curative intent was delivered using daily, conventionally fractionated (1.8-2 Gy per fraction) intensity-modulated RT (IMRT) to a total dose of 59.4-66 Gy. A clinical target volume (CTV) was defined as a 5 mm expansion from the gross tumor, adjusted for critical structures. The planning target volume (PTV) was a 3 mm expansion of the CTV. Planning goals included a prescription dose encompassing at least 95% of the PTV, no more than 20% of PTV receiving >110% of the prescription dose, no more than 1% of PTV receiving <93% of the prescription, and no more than 1% or 1 cc of tissue outside the PTV receiving >110% of the highest prescription dose. The cumulative spinal cord dose was limited to 54 Gy. In the event of a grade 3 or greater acute local toxicity, RT was to be held until grade 2 or lower was attained, or up to two weeks. In the event of an RT treatment break, chemotherapy was to be held until resumption of RT. Missed treatments due to acute toxicity or other non-oncologic reasons (scheduling, weather, transportation, or holidays) were to be made up; no modification of the ultimate RT dose was made.

Concurrent erlotinib was delivered daily throughout reirradiation at a dose of 100-150 mg, increasing by 25 mg increments (phase I) to the MTD, which was continued in phase II. Maintenance erlotinib was continued daily at a dose of 150 mg (non-smokers) to 300 mg (smokers) until progression, unacceptable toxicity, or two years. Pemetrexed 500 mg/m^2^ was delivered concurrently with reirradiation every 21 days. Oral folic acid (350-1000 mcg) was to be given daily beginning five to seven days prior to the first dose of pemetrexed and until three weeks after the last dose. Intramuscular vitamin B12 (1000 mcg) was administered approximately one week prior to the first dose of pemetrexed and repeated approximately every nine weeks until three weeks after the last dose. Dexamethasone (4 mg orally) was given twice daily on the day before, the day of, and the day after each dose of pemetrexed unless medically contraindicated.

The Functional Assessment of Cancer Therapy-Head and Neck (FACT-HN), the MD Anderson Dysphagia Inventory (MDADI), and the Performance Status Scale for Head and Neck Cancer (PSS-HN) were performed at baseline and 1, 6, 12, and 24 months from completion of RT [13-15]. The primary objectives of phase I were to evaluate the toxicity and determine the MTD of concurrent erlotinib. The primary objective of phase II was one-year PFS. Secondary objectives included best overall response (BOR, RECIST 1.1), OS, AEs per CTCAE version 3.0, and change in QOL from baseline to one year.

The phase I study aimed to determine the MTD of concurrent daily erlotinib with reirradiation using a 3+3 design. PFS and OS were calculated using the Kaplan-Meier method. AEs deemed at least possibly related to protocol therapy were summarized. We used Wilcoxon Signed Rank tests to assess the change in QOL from baseline to six months after RT. Cases with missing PRO data were excluded from individual analyses; missing data were not imputed or assumed to be missing at random. In 2016, the study was closed prior to meeting its goal of 35-40 patients; descriptive/exploratory analyses of the enrolled patients are presented. Analyses were performed in SAS 9.4. Two-sided P-values <0.05 were considered statistically significant.

## Results

A total of 27 patients were enrolled between January 2009 and May 2016: 15 in phase I and 12 in phase II. Baseline patient/disease characteristics and details on prior treatment and R/SP disease are presented in Table 1. Most patients were male, current/former smokers, with recurrent HNSCC and a recursive partitioning analysis (RPA) classification of II [16]. Prior RT dose ranged from 50 to 70 Gy. Of the 22 patients for whom disease status at reirradiation was known, 13 (49%) had recurrent disease and 9 (33%) had a second primary malignancy. The median time from prior RT to reirradiation was nine years (0.6-30). The site of reirradiation was a mucosal primary site in 16 (59%) patients and the neck in six patients (22%). Eighteen patients received 60 Gy, four received 66 Gy, and the median tumor volume was 28 cm^3^.

**Table 1:** Patient and treatment characteristics Data are summarized using n (%) or median (range) unless otherwise specified. ECOG: Eastern Cooperative Oncology Group; HPV: human papillomavirus; IMRT: intensity-modulated radiotherapy; MIRI: multi-institutional reirradiation [16]; RPA: recursive partitioning analysis; RT: radiotherapy; GTV: gross tumor volume.

Variable	Value
Age (years)	64 (51-77)
Sex	
Male	20 (74)
Female	7 (26)
Race	
White	25 (93)
Black/African American	1 (4)
Missing	1 (4)
Ethnicity	
Non-Hispanic	27 (100)
Hispanic	0 (0)
ECOG Performance Status	
0	6 (22)
1	21 (78)
Smoking History	
Never	7 (26)
Former	14 (52)
Current	2 (7)
Missing	4 (15)
Original Tumor Site	
Oral cavity	2 (7)
Oropharynx	8 (30)
Larynx	9 (33)
Hypopharynx	2 (7)
Unknown primary	1 (4)
Missing	5 (19)
Prior RT dose (n = 15)	
66.0 (50.0-70.2)	
Prior Surgery for Original Tumor	
Yes	15 (56)
Missing	5 (19)
Reirradiation Site	
Oral cavity	1 (4)
Oropharynx	13 (48)
Hypopharynx	2 (7)
Neck	6 (22)
Unknown	5 (19)
HPV Status of Reirradiated Site (n = 13)	
Positive	3 (23)
Negative/Unknown	10 (77)
Disease Status at Reirradiation	
Recurrence	13 (49)
Second primary	9 (33)
Missing	5 (19)
MIRI RPA Class	
II	19 (70)
III	2 (11)
Missing	5 (19)
Reirradiation Dose (Gy)	
59.4-60 Gy	18 (67)
66 Gy	4 (15)
Missing	5 (19)
Reirradiation GTV Volume (cm³, n = 21)	
28.1 (5.1-115.0)	
Erlotinib Dose Level (mg)	
100	4 (15)
125	18 (67)
150	5 (19)

No dose-limiting toxicities (DLTs) were observed at erlotinib doses of 100 and 125 mg; two DLTs were observed at 150 mg (grade 3 increase in liver function tests and grade 5 mesenteric ischemia leading to sepsis and death). The MTD was determined to be 125 mg, which was employed in phase II. The most common grade 3+ AEs observed in the phase I component of the study were lymphopenia, neutropenia, diarrhea, and metabolic abnormalities (Table 2). Overall, grade 3+ AEs occurred in 25/27 patients and comprised mostly of cytopenia, mucositis, diarrhea, and metabolic (Table 3). One grade 4 pulmonary hemorrhage occurred. There were two grade 5 toxicities: mesenteric ischemia and unspecified death (cause unknown).

**Table 2:** Summary of adverse events at least possibly related to treatment for the phase I trial component

Adverse Event	Number of Subjects, by CTCAE Version 3.0				
	Grade 3	Grade 4	Grade 5	Total	% Patients
Any Toxicity	14	4	1	14	93%
Blood/Bone Marrow	14	3	0	14	93%
Hemoglobin	4	1	0		
Leukocytes	13	2	0		
Neutrophils	7	3	0		
Platelets	1	0	0		
Cardiac: Arrhythmia	1	0	0	1	7%
Supraventricular arrhythmia	1	0	0		
Cardiac: General	2	0	0	2	13%
Hypotension	2	0	0		
Constitutional Symptoms	1	0	0	1	7%
Fatigue	1	0	0		
Dermatology/Skin	3	0	0	3	20%
Acne	1	0	0		
Radiation dermatitis	1	0	0		
Rash	1	0	0		
Gastrointestinal	5	0	1	5	33%
Anorexia	1	0	0		
Dehydration	1	0	0		
Diarrhea	4	0	0		
Dysphagia	1	0	0		
GI - Other	0	0	1		
Nausea	1	0	0		
Vomiting	1	0	0		
Infection	3	0	0	3	20%
Febrile neutropenia	1	0	0		
Infection (documented clinically) with grades 3 or 4 absolute neutrophil count	1	0	0		
Infection with normal absolute neutrophil count	2	0	0		
Lymphatics	1	0	0	1	7%
Edema: head and neck	1	0	0		
Metabolic/Laboratory	6	1	0	6	40%
Alanine aminotransferase increased	1	0	0		
Aspartate aminotransferase increased	2	0	0		
Albumin, serum-low	3	0	0		
Hyperglycemia	1	0	0		
Hypernatremia	1	0	0		
Hypocalcemia	2	1	0		
Hypokalemia	1	0	0		
Hyponatremia	1	0	0		
Hypophosphatemia	3	0	0		
Pain	1	0	0	1	7%
Pain	1	0	0		
Pulmonary/Upper Respiratory	1	0	0	1	7%
Aspiration	1	0	0		

**Table 3:** Summary of adverse events at least possibly related to treatment for the combined phase I/II trial

Adverse Event	Number of Subjects, by CTCAE Version 3.0				
	Grade 3	Grade 4	Grade 5	Total	% Patients
Any Toxicity	25	8	2	25	93%
Blood/Bone Marrow	25	6	0	25	93%
Hemoglobin	6	2	0		
Leukocytes	23	4	0		
Neutrophils	10	4	0		
Platelets	1	0	0		
Cardiac: Arrhythmia	1	0	0	1	4%
Supraventricular arrhythmia	1	0	0		
Cardiac: General	2	0	0	2	7%
Hypotension	2	0	0		
Constitutional Symptoms	1	0	0	1	4%
Fatigue	1	0	0		
Death	0	0	1	1	4%
Dermatology/Skin	5	0	0	5	19%
Acne	2	0	0		
Radiation dermatitis	1	0	0		
Rash	2	0	0		
Gastrointestinal	9	0	1	9	33%
Anorexia	1	0	0		
Dehydration	1	0	0		
Diarrhea	4	0	0		
Dysphagia	2	0	0		
GI - Other	0	0	1		
Mucositis	4	0	0		
Nausea	1	0	0		
Vomiting	1	0	0		
Hemorrhage/Bleeding	0	1	0	1	4%
Hemorrhage pulmonary	0	1	0		
Infection	4	0	0	4	15%
Febrile neutropenia	2	0	0		
Infection (documented clinically) with grades 3 or 4 absolute neutrophil count	1	0	0		
Infection with normal absolute neutrophil count	2	0	0		
Lymphatics	1	0	0	1	4%
Edema: head and neck	1	0	0		
Metabolic/Laboratory	7	1	0	7	26%
Alanine aminotransferase increased	1	0	0		
Aspartate aminotransferase increased	2	0	0		
Albumin, serum-low	3	0	0		
Hyperglycemia	1	0	0		
Hypernatremia	1	0	0		
Hypocalcemia	2	1	0		
Hypokalemia	1	0	0		
Hyponatremia	2	0	0		
Hypophosphatemia	3	0	0		
Pain	2	0	0	2	7%
Pain	2	0	0		
Pulmonary/Upper Respiratory	2	0	0	2	7%
Aspiration	1	0	0		
Hypoxia	1	0	0		

**Figure 1:**
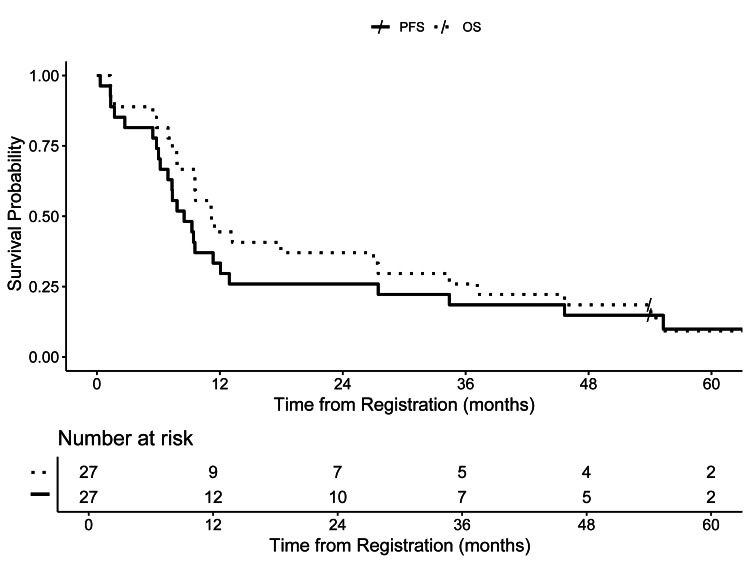
Progression-free survival and overall survival

Of 22 patients with available data, 19 (86%) completed prescribed RT. BOR was complete (n = 7), partial (n = 7), stable (n = 5), and progressive (n = 4), with four patients not evaluable. Estimates of one-year PFS and OS were 33.3% (95% CI: 16.8-50.9) and 44.4% (25.6-61.7), respectively (Figure 1). Median PFS and OS were 8.5 (95% CI: 6.0-12.1) and 11.2 months (7.8-27.5), respectively.

**Figure 2:**
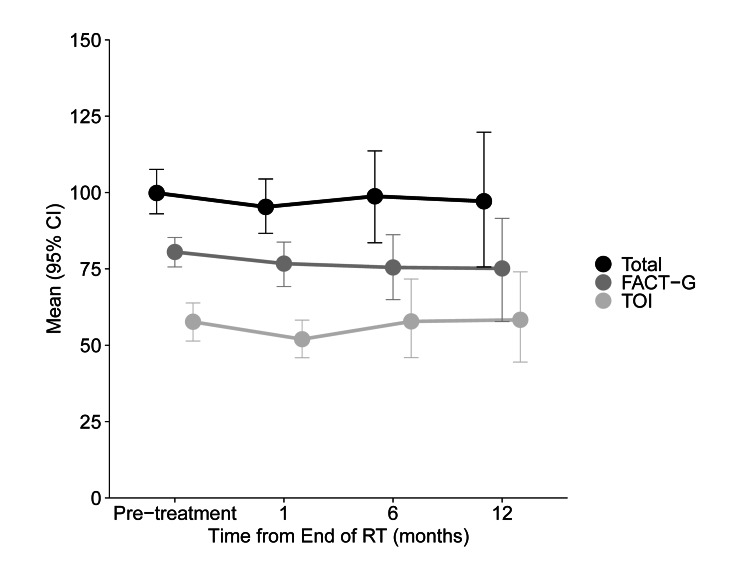
Mean FACT-HN total, FACT-G, and trial outcome index (TOI) scores over time Error bars indicate standard error. FACT-HN: Functional Assessment of Cancer Therapy-Head and Neck; FACT-G: Functional Assessment of Cancer Therapy-General.

**Figure 3:**
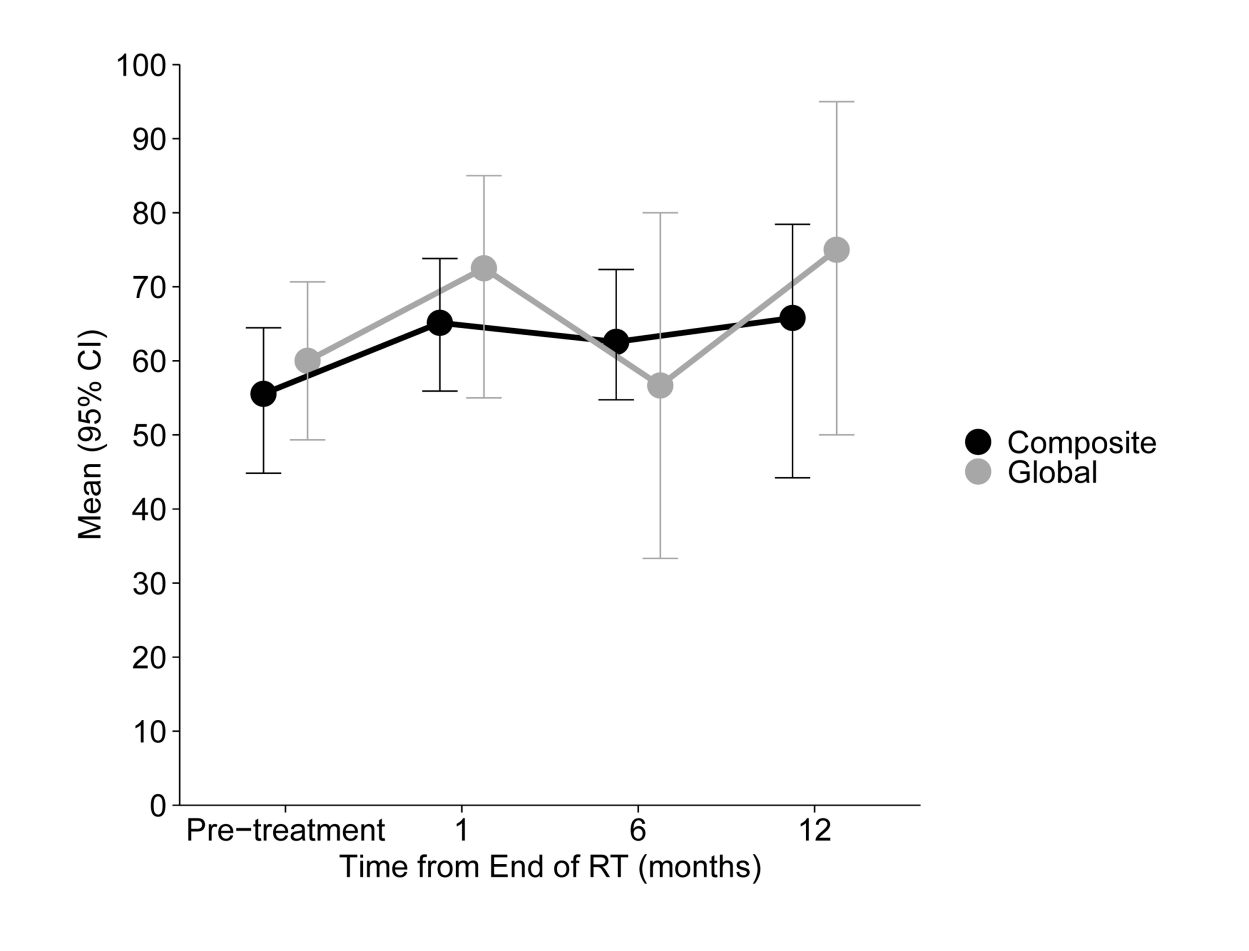
Mean MDADI scores over time Error bars indicate standard error. MDADI: MD Anderson Dysphagia Inventory.

No differences in any QOL scores (FACT-HN, MDADI, or PSS-HN) were observed from baseline to six months (Table 4). FACT-HN scores are displayed in Figure 2 and summarized in Table 5. There were no differences in total FACT-HN as well as Functional Assessment of Cancer Therapy-General (FACT-G) and trial outcome index (TOI) scores over time. MDADI global, composite, and subscale scores are displayed in Figure 3 and summarized in Table 6. Similarly, mean MDADI scores did not differ over time (Table 2). No differences in normalcy of diet, public eating, or understandability of speech measured by the PSS-HN were observed over time (Table 7).

**Table 4:** Comparison of FACT-HN, MDADI, and PSS-HN scores from baseline to six months after reirradiation P-values are from paired t-tests between pre-treatment and one-year scores. FACT-HN: Functional Assessment of Cancer Therapy-Head and Neck; MDADI: MD Anderson Dysphagia Inventory; PSS-HN: Performance Status Scale for Head and Neck Cancer.

Instrument	Pre-treatment		Six months		P-value
	n	Mean (95% CI)	n	Mean (95% CI)	
FACT-HN					
Trial Outcome Index	22	57.7 (51.1, 64.2)	9	57.8 (42.1, 73.5)	0.578
FACT-G Total Score	22	80.5 (75.0, 86.1)	10	75.5 (61.9, 89.0)	0.469
FACT-HN Total Score	22	99.8 (92.2, 107.5)	9	98.8 (79.7, 117.8)	0.578
MDADI					
Global score	15	60.0 (47.4, 72.6)	6	56.7 (20.5, 92.8)	Inestimable
Composite score	17	55.5 (44.3, 66.8)	7	63.3 (49.5, 77.1)	0.688
Emotion score	15	70.2 (63.6, 76.9)	7	62.9 (48.7, 77.0)	0.625
Function score	15	64.3 (57.7, 70.8)	7	61.8 (47.8, 75.8)	1.000
Physical score	15	56.7 (50.3, 63.1)	7	62.6 (50.3, 74.9)	1.000
PSS-HN					
Normalcy of diet	16	46.9 (26.9, 66.9)	7	55.7 (7.4, 104.0)	1.000
Eating in public	15	60.0 (42.0, 78.0)	7	64.3 (34.9, 93.7)	1.000
Understandability of speech	15	71.7 (54.4, 88.9)	7	67.9 (42.1, 93.6)	1.000

**Table 5:** Mean (95% CI) scores for the FACT-HN at each time point Results are displayed as estimate (95% CI). Scores range 0-148 for the FACT-HN total score, 0-108 for the FACT-G, 0-96 for the TOI, 0-28 for PWB, SWB, and FWB subscales, 0-24 for the EWB subscale, and 0-40 for the HNCS. P-values are from repeated measures models testing for a change in scores over time. Higher scores indicate better QOL. FACT-HN: Functional Assessment of Cancer Therapy-Head and Neck; FACT-G: Functional Assessment of Cancer Therapy-General; TOI: trial outcome index; PWB: Physical Well-Being; SWB: Social Well-Being; EWB: Emotional Well-Being; FWB: Functional Well-Being; HNCS: Head and Neck Cancer Subscale; QOL: quality of life.

Domain	Pre-treatment	One-Month Follow-up	Six-Month Follow-up	One-Year Follow-up	P-value
Total (n)	22	13	10	6	-
FACT-HN					
FACT-HN total score	99.1 (90.3, 107.8)	92.8 (82.5, 103.2)	96.3 (84.5, 108.1)	90.5 (77.0, 104.0)	0.4421
FACT-G total score	79.4 (72.8, 86.1)	75.5 (67.9, 83.2)	75.9 (67.6, 84.2)	71.5 (61.9, 81.1)	0.3017
TOI	57.5 (50.6, 64.4)	49.8 (41.5, 58.2)	55.2 (45.6, 64.8)	52.9 (41.8, 63.9)	0.2755
FACT-HN Subscales					
Physical Well-Being (PWB)	21.1 (18.8, 23.5)	17.8 (15.0, 20.6)	18.7 (15.7, 21.8)	18.8 (15.1, 22.4)	0.0713
Social Well-Being (SWB)	24.7 (23.0, 26.4)	24.6 (22.6, 26.6)	24.1 (21.8, 26.3)	22.6 (19.9, 25.2)	0.3546
Emotional Well-Being (EWB)	16.9 (14.7, 19.1)	18.4 (15.8, 20.9)	16.3 (13.5, 19.1)	15.0 (11.7, 18.3)	0.1620
Functional Well-Being (FWB)	16.9 (14.3, 19.4)	14.8 (11.7, 18.0)	16.4 (13.0, 19.9)	15.3 (11.1, 19.6)	0.5848
Head and Neck Cancer Subscale (HNCS)	19.5 (15.9, 23.1)	16.9 (12.7, 21.1)	19.8 (15.2, 24.5)	18.3 (13.1, 23.5)	0.3833

**Table 6:** Mean (95% CI) scores for the MDADI at each time point Higher scores indicate better swallowing-related QOL. MDADI: MD Anderson Dysphagia Inventory; QOL: quality of life.

Domain	Pre-treatment	One-Month Follow-up	Six-Month Follow-up	One-Year Follow-up	P-value
Total (n)	17	8	7	4	-
Global score	59.5 (45.3, 73.7)	71.3 (52.7, 89.9)	60.7 (39.5, 82)	65.8 (40.3, 91.3)	0.6285
Composite score	56.0 (46.5, 65.6)	64.5 (52.1, 77.0)	65.5 (52.3, 78.7)	64.6 (48.1, 81.0)	0.3324
Emotion score	69.5 (61.4, 77.6)	72.1 (61.6, 82.5)	66.6 (55.4, 77.8)	64.8 (50.6, 79.1)	0.7361
Function score	63.5 (54.4, 72.6)	64.6 (52.4, 76.7)	65.0 (51.9, 78)	65.5 (48.6, 82.4)	0.9938
Physical score	56.7 (49.4, 64.1)	61.6 (52.5, 70.7)	62.8 (53.2, 72.5)	61.4 (49.5, 73.2)	0.5025

**Table 7:** Mean (95% CI) scores for the PSS-HN at each time point Results are displayed as an estimate (95% CI). Scores range from 0 to 100 for all scales. P-values are from repeated measures models testing for a change in scores over time. PSS-HN: Performance Status Scale for Head and Neck Cancer.

Domain	Pre-treatment	One-Month Follow-up	Six-Month Follow-up	One-Year Follow-up	P-value
Total (n)	16	11	7	3	-
Normalcy of Diet	48.5 (28.4, 68.6)	33.5 (10.8, 56.3)	54.8 (27.7, 81.8)	58.4 (20.8, 96.1)	0.2637
Eating in Public	60.1 (42.6, 77.6)	46.2 (25.4, 67.0)	62.7 (38.3, 87.1)	69.1 (33.4, 104.8)	0.4165
Understandability of Speech	69.1 (53.1, 85.1)	59.7 (42.3, 77.1)	66.8 (46.7, 86.9)	87.1 (60.3, 113.9)	0.1397

## Discussion

In this study, we describe the disease control, toxicity, and QOL outcomes for patients treated with reirradiation with concurrent pemetrexed/erlotinib and maintenance erlotinib. We observed PFS and OS outcomes that were fairly consistent with early reirradiation trials, which demonstrated median PFS estimates of approximately eight months, median OS 12 months, and one-year survival rates ranging from 23% to 50% [7,17,18]. We also describe PRO measures of QOL as qualitatively stable through the first year after treatment.

Although the selection of the combination of systemic treatments utilized in this study is based on earlier data, there is currently a renewed interest in EGFR inhibitors for the management of HNSCC [19]. Exploiting their demonstrated radiosensitizing effect and favorable clinical tolerance supported the hypothesis for an improved toxicity profile of reirradiation with this drug combination. These results do not support additional prospective study of this specific treatment paradigm, especially within the current landscape of systemic therapy for patients with R/SP HNSCC. Instead, the importance of this study lies in its comprehensive assessment of QOL in this patient population. In initial studies of reirradiation for patients with limited survival treated with high-risk curative-intent therapies, QOL assessment may not have been reasonable [4,7,20]. Over the years, oncology research has better reflected our patients’ wishes to live longer, better lives with an increased focus on QOL in addition to disease control and survival. While it remains the case that patients with R/SP HNSCC often accept high risks of severe toxicity with curative reirradiation, this should not negate the utility of PRO measures.

Our findings are in line with the few prospective studies to collect and report QOL outcomes. A promising phase II trial of reirradiation with concurrent and adjuvant nivolumab observed no significant differences in FACT-HN scores over time [5]. Qualitatively, PWB and SWB subscale scores were slightly higher over time than EWB and FWB scores, and FACT-G scores ranged from 70 to 80, consistent with our observations. An observational study measured EORTC QLQ-C30 and H&N35 in patients treated with reirradiation and found that most patients experienced stable patient-reported QOL at 3 and 12 months [21]. Similar to our study, attrition was high at later time points. Analysis of three-year survivors identified numeric but non-significant worsening of multiple functional and symptom scores. Such measures, similar to those employed, developed primarily in patients without prior treatment, may have more limited sensitivity to detect QOL decrements in patients with R/SP disease treated with reirradiation. A response shift bias due to the pre-existing burden of disease and treatment effects could impact their utility in this heavily pre-treated patient population.

Our findings expand the published QOL data for patients treated with curative reirradiation for HNSCC by reporting additional measures, including MDADI and PSS-HN, up to one year after RT. Mean MDADI composite scores remained stable between baseline and one year (mean 56 to 65). These values are on par with scores reported from patients treated with a single course of RT for de novo disease and are slightly above the threshold of “clinically acceptable swallowing” (MDADI composite score of 60 or greater) set by NRG HN002 [22]. PSS-HN generally remained unchanged over time.

This study is limited by early closure and small sample size, and high attrition rates early in follow-up limited our statistical power. Given the time since original data collection occurred, institutional standards regarding missing data during that period, and the lack of a prospective framework for identifying reasons for missingness in PRO data, we were unable to ascertain the specific reasons for missing data. Thus, we report herein all protocol-specified descriptive analyses of disease and QOL. Also, source data on systemic therapy adherence are unavailable.

## Conclusions

In conclusion, patient-reported QOL in patients treated with reirradiation for R/SP HNSCC remains stable in the first year of follow-up, within which progression is frequent. Increased focus on PROs of QOL after reirradiation is needed to better understand and measure performance in this population, inform patient selection, and optimize shared decision-making.
